# Effect of a structured early rehabilitation program on long-term functional recovery, quality of life, and survival in patients with severe acute pancreatitis: a randomized controlled trial

**DOI:** 10.1186/s12876-026-04955-7

**Published:** 2026-05-27

**Authors:** Li Wang, Ziqi Nie, Qiandi Huang

**Affiliations:** 1 Department of Comprehensive Healthcare, Zhongnan Hospital of Wuhan University, Wuhan, Hubei China; 2 Department of General Practice, The Sixth Hospital of Wuhan, Affiliated Hospital of Jianghan University, Wuhan, Hubei China; 3 Scientific Research Office, The Sixth Hospital of Wuhan, Affiliated Hospital of Jianghan University, No. 168 Hongkong Road, Jianghan District, Wuhan, Hubei Province 430000 China

**Keywords:** Early rehabilitation nursing, Severe acute pancreatitis, Critical care, Long-term follow-up, Functional recovery, Quality of life

## Abstract

**Background:**

Prolonged immobilization in patients with severe acute pancreatitis (SAP) leads to profound physical deconditioning, yet structured rehabilitation protocols remain underexplored in this population.

**Objective:**

To investigate the effects of a structured early rehabilitation nursing (ERN) program on short-term and long-term clinical outcomes, including length of stay, functional recovery, and survival, in patients with severe acute pancreatitis (SAP) admitted to the intensive care unit (ICU).

**Methods:**

This single-center, prospective, randomized controlled trial was conducted from June 2019 to June 2024. A total of 400 eligible SAP patients admitted to the intensive care unit (ICU) were randomized (1:1) to either the ERN group (*n* = 200), receiving a protocolized, multidisciplinary rehabilitation program, or the control group (*n* = 200), receiving usual care. The primary outcome was hospital length of stay (LOS). Key secondary outcomes included ICU LOS, systemic inflammatory markers (CRP, IL-6), muscle strength (MRC sum score), activities of daily living (ADL), quality of life (WHOQOL-BREF), and 1-year all-cause mortality. Assessments were performed at baseline, discharge, and at 3, 6, and 12 months post-discharge.

**Results:**

The ERN group had significantly shorter hospital LOS (11.5 ± 2.5 vs. 14.8 ± 3.1 days, *P* < 0.001) and ICU LOS (7.2 ± 2.1 vs. 10.5 ± 2.8 days, *P* < 0.001). At day 7, the ERN group showed lower levels of CRP (48.5 ± 15.2 vs. 95.3 ± 25.4 mg/L, *P* < 0.001) and IL-6 (55.2 ± 18.3 vs. 110.6 ± 30.1 pg/mL, *P* < 0.001). At discharge, the ERN group demonstrated superior muscle strength (MRC score: 52.4 ± 4.9 vs. 46.1 ± 5.8, *P* < 0.001) and functional independence (ADL score: 75.8 ± 8.1 vs. 62.5 ± 9.3, *P* < 0.001). These functional and quality of life benefits were sustained through the 12-month follow-up. Kaplan-Meier analysis revealed a significantly higher 1-year survival rate in the ERN group (95.0% vs. 87.5%; log-rank *P* = 0.012). The incidence of ICU-acquired complications was also lower in the ERN group (5.5% vs. 26.0%, *P* < 0.001).

**Conclusion:**

A structured, early rehabilitation nursing program for SAP patients effectively attenuates systemic inflammation, accelerates physical recovery, reduces length of stay, and improves long-term functional outcomes, quality of life, and 1-year survival. These findings support the integration of ERN as a standard of care in the management of critically ill SAP patients.

**Supplementary Information:**

The online version contains supplementary material available at 10.1186/s12876-026-04955-7.

## Introduction

Severe acute pancreatitis (SAP) is a critical inflammatory disorder of the pancreas associated with a high risk of systemic inflammatory response syndrome (SIRS), multi-organ dysfunction syndrome (MODS), and substantial mortality, which can reach up to 30% in severe cases [1, 2]. The management of SAP in the intensive care unit (ICU) has traditionally focused on aggressive fluid resuscitation, nutritional support, and organ support [3]. However, patients with SAP are often subjected to often require prolonged periods of bed rest and mechanical ventilation. This prolonged immobilization is frequently driven not only by the acute physiological derangements of the disease but also by traditional ICU unit cultures and practitioner behaviors that favor deep sedation and conservative management [4]. This inactivity precipitates a cascade of deleterious effects, including ICU-acquired weakness (ICU-AW), diaphragmatic dysfunction, venous thromboembolism, and delayed gastrointestinal recovery [4–6]. These complications not only prolong ICU and hospital stays but also lead to significant long-term functional impairment, reduced quality of life (QoL), and increased healthcare costs [7, 8].

While early mobilization has shown short-term benefits in general critical care populations by mitigating the adverse effects of immobilization [8, 9], its longer-term functional benefits remain less conclusive. Recent scoping reviews highlight significant methodological challenges in the existing literature, including inadequate reporting of mobilization doses (e.g., intensity and progression) [10] and high heterogeneity or poor description of comparator groups (often labeled simply as “usual care”) [11]. Despite these limitations, the principle of early rehabilitation, initiated within the first 48–72 h of ICU admission, is to preserve muscle mass, enhance respiratory function, and promote functional independence [12]. For SAP patients specifically, early rehabilitation may offer unique advantages by improving splanchnic circulation, modulating the systemic inflammatory response, and facilitating earlier enteral nutrition tolerance [13]. However, the implementation of early rehabilitation for SAP is often hampered by concerns over safety, such as hemodynamic instability, intra-abdominal hypertension, and the risk of dislodging percutaneous drains [14].

While some studies have explored the benefits of physical therapy or accelerated care pathways in Severe Acute Pancreatitis (SAP), they are often limited by small sample sizes and focus almost exclusively on short-term hospital-based outcomes, neglecting long-term quality of life and functional recovery [15, 16]. Specifically, recent evidence confirms that the literature on the management of SAP predominantly comprises studies on acute intensive care unit (ICU) interventions and immediate complications (e.g., organ failure resolution), with minimal data on post-discharge outcomes such as pancreatic function, psychological impact, or patient-reported quality of life metrics [15]. This gap persists despite calls for comprehensive assessments incorporating longitudinal morbidity beyond the acute phase [17]. The long-term impact on functional status, sustained QoL, and mortality remains poorly defined. Furthermore, existing protocols often neglect crucial components of holistic rehabilitation, such as structured respiratory muscle training, systematic psychological support, and patient-centered goal setting, which are vital for comprehensive recovery [18]. This study aims to address these critical gaps by evaluating the efficacy of a comprehensive, structured early rehabilitation nursing (ERN) program in a large cohort of SAP patients. We hypothesized that this multifaceted intervention, compared to usual care, would not only accelerate in-hospital recovery and reduce complications but also lead to superior long-term functional independence, QoL, and survival.

## Materials and methods

### Study design and participants

**Fig. 1:**
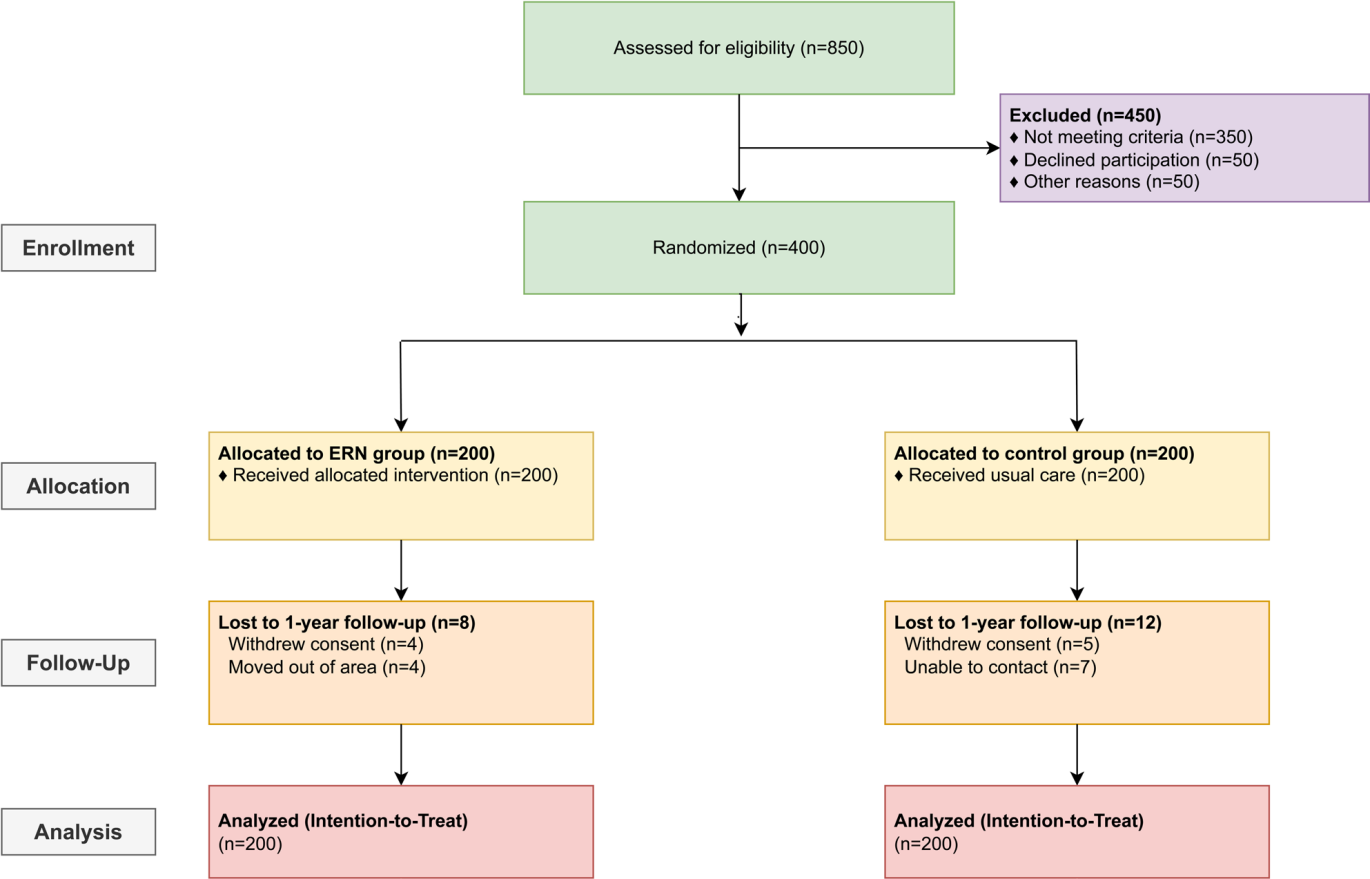
The consort 2010 flow diagram. The diagram details the flow of participants through each stage of the randomized trial, from assessment for eligibility to randomization, allocation, follow-up, and analysis

This study was a single-center, prospective, parallel-group, randomized controlled trial conducted at the ICU of The Sixth Hospital of Wuhan, a tertiary academic medical center in China. The trial was approved by the Institutional Review Board and Ethics Committee of The Sixth Hospital of Wuhan (Approval Date: March 28, 2019). Written informed consent was obtained from all participants or their legal surrogates prior to randomization. The study was conducted in accordance with the Declaration of Helsinki and reported following the CONSORT 2010 statement [19] (Fig. 1). This study was retrospectively registered with ClinicalTrials.gov (Identifier: NCT07109921; Registration Date: July 30, 2025). The delay in registration was due to an administrative oversight, but the protocol was approved prior to recruitment and strictly followed.

Patients were recruited from June 2019 to June 2024. Inclusion criteria were: (1) age 18 to 75 years; (2) a diagnosis of SAP according to the 2012 revised Atlanta classification [20]; (3) admission to the ICU within 72 h of symptom onset; (4) an anticipated ICU stay of more than 48 h. Exclusion criteria were: (1) pre-existing conditions precluding rehabilitation (e.g., severe neuromuscular disease, unstable spinal fractures); (2) acute myocardial infarction or unstable cardiac arrhythmia; (3) intracranial hypertension; (4) active gastrointestinal bleeding; (5) pregnancy; or (6) patient or family refusal.

### Randomization and blinding

Eligible patients were randomly assigned in a 1:1 ratio to either the ERN group or the control group using a computer-generated randomization sequence with permuted blocks of varying sizes (4 and 6). The allocation sequence was concealed in sequentially numbered, opaque, sealed envelopes. Due to the nature of the intervention, blinding of patients and the clinical staff delivering the rehabilitation was not feasible. However, outcome assessors and data analysts were blinded to group allocation.

### Interventions

#### Control group (usual care)

Patients in the control group received standard ICU care for SAP, which included hemodynamic monitoring, mechanical ventilation, fluid management, nutritional support, pain control, and supportive care as directed by the primary ICU team. Rehabilitation was not protocolized and was provided at the discretion of the primary ICU physicians. In this usual care setting, the involvement of physical therapists during the acute ICU phase was minimal; mobility interventions were typically limited to passive range of motion exercises performed occasionally by bedside nurses, without a structured multidisciplinary progression plan.

#### ERN group (structured early rehabilitation nursing program)

In addition to usual care, patients in the ERN group received a protocolized, multidisciplinary rehabilitation program initiated within 48 h of ICU admission, provided they met safety criteria (e.g., hemodynamic stability, adequate oxygenation). The program was delivered by a dedicated multidisciplinary team comprising ICU physicians, trained ICU nurses, and physical therapists, working collaboratively 7 days a week. All team members underwent a standardized 2-week training module covering early mobilization safety criteria, multidisciplinary exercise progression, and protocol fidelity. The protocol (detailed in Supplementary Table 1) included: (1) early mobilization starting with passive range of motion and progressing to active ambulation; (2) respiratory training including deep breathing and incentive spirometry; and (3) structured psychological support.

The structured phases of this mobility protocol were adapted from the foundational early ICU mobility therapy framework developed by Morris et al. [21]. The program was structured in phases: Phase 1 (Unconscious or Deep Sedation): Passive range of motion exercises (20 min, twice daily), regular turning and repositioning every 2 h, and maintenance of optimal limb positioning. Phase 2 (Awake and Hemodynamically Stable): Respiratory Training: Deep breathing exercises and incentive spirometry (10 breaths per hour while awake). Bed Mobility: Active-assisted range of motion, bridging exercises, rolling. Progressive Mobilization: Gradual head-of-bed elevation, progressing to sitting at the edge of the bed (20–30 min, 1–2 times daily), and transfer to a chair. Phase 3 (Active Mobilization): In-place stepping, progressing to supervised ambulation with a walker. The goal was to walk at least 10 m, 2–3 times daily. Psychological Support: Rather than ad-hoc encouragement, a structured cognitive-behavioral intervention was delivered by trained ICU nurses for 15 min daily. This standardized protocol included 5 min of orienting communication, 5 min of systematic goal-setting, and 5 min of progressive muscle relaxation and deep-breathing exercises tailored to manage anxiety and delirium symptoms.

Safety criteria were monitored before and during each session, utilizing specific thresholds for pausing or modifying intervention (e.g., SpO2 < 90%, heart rate < 40 or > 130 bpm, mean arterial pressure < 65 mmHg, or requirement of norepinephrine equivalent dose ≥ 0.1 µg/kg/min). Following discharge from the ICU to the general ward, rehabilitation care was no longer protocolized for either group and was provided at the discretion of the ward-based clinical team. All patients received a standardized discharge package encouraging physical activity, but no formal outpatient rehabilitation was mandated for either group.

### Outcomes and measurements

#### Primary outcomes

The primary outcome was hospital length of stay (LOS), defined as the total time from hospital admission to discharge.

#### Secondary outcomes

Key secondary outcome was ICU length of stay (LOS). Other secondary outcomes included: Inflammatory Markers: Serum levels of C-reactive protein (CRP) and Interleukin-6 (IL-6) were measured at baseline and on day 7. Muscle Strength: Assessed at ICU discharge using the Medical Research Council (MRC) sum score, which evaluates strength in six muscle groups bilaterally (range 0–60). A score < 48 indicates ICU-AW [22]. Functional Independence: Assessed using the Barthel Index for Activities of Daily Living (ADL) via direct observation by blinded assessors at baseline and ICU discharge, and through structured telephone interviews with the patients or their primary caregivers at 3, 6, and 12 months post-discharge (range 0–100, higher scores indicate greater independence) [23]. Quality of Life: Measured using the World Health Organization Quality of Life-BREF (WHOQOL-BREF) instrument at the same time points as the ADL. It comprises four domains: physical health, psychological, social relationships, and environment [24]. One-Year Survival: All-cause mortality was tracked for 12 months after randomization through hospital records and telephone follow-up. Safety Outcomes: The incidence of pre-specified ICU-acquired complications (pressure ulcers, ventilator-associated pneumonia, deep vein thrombosis) was recorded. Additionally, adverse events during rehabilitation sessions (e.g., hemodynamic instability, desaturation) and device dislodgements were monitored. Patient Satisfaction with Multidisciplinary Care: Patient and/or family satisfaction with the overall care provided by the multidisciplinary team (including both nursing and physical therapy services) was assessed at discharge. This was measured using a validated 5-point Likert scale adapted from the Patient Satisfaction with Nursing Care Quality Questionnaire (PSNCQQ) [25] to reflect the broader scope of multidisciplinary rehabilitation providers.

### Statistical analysis

The sample size was calculated based on the primary outcome of hospital LOS. Based on our pilot data and previous literature [26], we anticipated a mean reduction of 2.5 days in hospital LOS with a standard deviation of 7.0 days. To detect this difference with 80% power at a two-sided alpha level of 0.05, a sample size of 170 patients per group was required. To account for a potential 15% attrition rate during the 1-year follow-up, we aimed to enroll a total of 400 patients (200 per group).

All analyses were performed on an intention-to-treat (ITT) basis. Missing data for long-term outcomes due to loss to follow-up were handled using multiple imputation. Specifically, missing data accounted for 5.0% of the total observations (20 out of 400 patients were lost to follow-up: 8 in the ERN group and 12 in the control group). Assuming the data were missing at random (MAR), multiple imputation by chained equations (MICE) was employed, generating 5 imputed datasets. The imputation model included baseline age, gender, APACHE II score, treatment group allocation, and baseline ADL and QoL scores. Continuous variables were presented as mean ± standard deviation (SD) or median (interquartile range, IQR) as appropriate. Categorical variables were presented as numbers (percentages). For baseline characteristics, differences between groups were assessed using independent t-tests or Mann-Whitney U tests for continuous variables and chi-squared or Fisher’s exact tests for categorical variables. For primary and secondary outcomes, linear regression was used to compare continuous outcomes, adjusting for baseline values. To account for potential confounding, adjusted mean differences for length of stay were calculated using multivariate linear regression adjusting for baseline continuous age and APACHE II scores. Comparisons of Day 7 inflammatory markers were performed using ANCOVA, adjusting for baseline values. Longitudinal data (ADL, QoL) were analyzed using a linear mixed-effects model with random intercepts to account for repeated measures, including group, time, and group-by-time interaction as fixed effects. Survival was analyzed using the Kaplan-Meier method, and Cox proportional hazards regression was used to calculate the adjusted hazard ratio (aHR) controlling for age and APACHE II score. A pre-planned subgroup analysis was conducted for hospital LOS based on baseline APACHE II score (< 15 vs. ≥15). A two-sided P-value < 0.05 was considered statistically significant. All analyses were performed using SPSS version 26.0 (IBM Corp., Armonk, NY, USA).

## Results

### Patient enrollment and characteristics

From June 2019 to June 2024, a total of 850 patients with SAP were assessed for eligibility. Of these, 450 were excluded (350 did not meet inclusion criteria, 50 declined to participate, and 50 were excluded for other reasons). The remaining 400 patients were randomized, with 200 allocated to the ERN group and 200 to the control group. During the 1-year follow-up, 8 patients (4.0%) in the ERN group and 12 (6.0%) in the control group were lost to follow-up. However, all 400 randomized patients were included in the ITT analysis for primary outcomes and survival. The detailed flow of participants is shown in Fig. 1. The baseline demographic and clinical characteristics were well-balanced between the two groups (Table 1).

**Table 1:** Baseline demographic and clinical characteristics of patients

Parameter	ERN Group (*n* = 200)	Control Group (*n* = 200)	Statistic (t/χ²)	*P*-value
Age (years), mean ± SD	52.5 ± 12.8	53.1 ± 13.2	-0.485	0.628
Gender (male), *n* (%)	122 (61.0%)	118 (59.0%)	0.161	0.688
Time from onset to ICU admission (hours)	48.5 ± 12.4	49.2 ± 13.1	-0.548	0.584
Etiology, *n* (%)				
Biliary	98 (49.0%)	91 (45.5%)	0.452	0.501
Hyperlipidemia	52 (26.0%)	58 (29.0%)	0.489	0.484
Alcohol	38 (19.0%)	42 (21.0%)	0.244	0.621
Other/Idiopathic	12 (6.0%)	9 (4.5%)	0.467	0.494
Comorbidities, *n* (%)				
Hypertension	61 (30.5%)	55 (27.5%)	0.428	0.513
Diabetes Mellitus	48 (24.0%)	42 (21.0%)	0.471	0.492
Severity Scores at Admission, mean ± SD				
APACHE II Score	14.2 ± 3.5	14.5 ± 3.8	-0.852	0.395
BISAP Score	2.9 ± 0.8	3.0 ± 0.9	-1.201	0.231
Marshall Score	3.1 ± 1.2	3.2 ± 1.1	-0.869	0.385
Balthazar CT Grade D/E, *n* (%)	90 (45.0%)	92 (46.0%)	0.040	0.841
Inflammatory Markers at Baseline, mean ± SD				
CRP (mg/L)	150.2 ± 45.3	148.5 ± 42.1	0.389	0.702
IL-6 (pg/mL)	220.5 ± 65.4	218.1 ± 62.8	0.375	0.715
Organ Failure Breakdown, *n* (%)				
Respiratory Failure (Invasive Mechanical Ventilation)	140 (70.0%)	138 (69.0%)	0.048	0.827
Renal Failure (CRRT)	30 (15.0%)	34 (17.0%)	0.301	0.583
Cardiovascular Failure (Vasopressors)	45 (22.5%)	48 (24.0%)	0.127	0.721
Local Complications, *n* (%)				
Acute Necrotic Collection / Walled-off Necrosis	85 (42.5%)	88 (44.0%)	0.091	0.763
Acute Peripancreatic Fluid Collection / Pseudocyst	60 (30.0%)	65 (32.5%)	0.292	0.589

Data are presented as mean ± standard deviation (SD) or *n* (%)

*Abbreviations*: *ERN* Early Rehabilitation Nursing, *SD* Standard Deviation, *ICU* Intensive Care Unit, *APACHE II* Acute Physiology and Chronic Health Evaluation II, *BISAP* Bedside Index of Severity in Acute Pancreatitis, *CT* Computed Tomography, *CRP* C-Reactive Protein, *IL-6* Interleukin-6, *CRRT* Continuous Renal Replacement Therapy

*P*-values were calculated using independent t-tests for continuous variables and χ² tests for categorical variables

### Adherence to intervention

Adherence to the ERN protocol was high. Of the 1,456 prescribed rehabilitation sessions for the ERN group (mean 7.28 sessions per patient), 1,340 (92.0%) were delivered. The mean duration of delivered sessions was 28.5 ± 5.2 min. The most common reasons for session cancellation were patient refusal or distress (e.g., severe pain, intense anxiety, or verbalized exhaustion; 4.5% of missed sessions) and hemodynamic or respiratory instability (3.5% of missed sessions).

### Primary outcomes

The total hospital LOS was significantly reduced in the ERN group (11.5 ± 2.5 days vs. 14.8 ± 3.1 days; difference, -3.3 days; 95% CI, -4.0 to -2.6; *P* < 0.001) (Table 2). After adjusting for baseline age and APACHE II scores, the hospital LOS remained significantly reduced (adjusted Mean Difference [aMD], -3.2 days; 95% CI, -3.9 to -2.5; *P* < 0.001). Patients in the ERN group also had a significantly shorter ICU LOS compared to the control group (7.2 ± 2.1 days vs. 10.5 ± 2.8 days; difference, -3.3 days; 95% CI, -3.9 to -2.7; *P* < 0.001). Furthermore, the duration of mechanical ventilation was significantly shorter in the ERN group (4.1 ± 1.9 vs. 6.8 ± 2.5 days, *P* < 0.001) (Table 2).

**Table 2:** Primary and key in-hospital outcomes

Outcome	ERN Group (*n* = 200)	Control Group (*n* = 200)	Difference / aMD (95% CI)	*P*-value
Hospital Length of Stay (days), mean ± SD	11.5 ± 2.5	14.8 ± 3.1	-3.2 (-3.9 to -2.5)*	< 0.001
ICU Length of Stay (days), mean ± SD	7.2 ± 2.1	10.5 ± 2.8	-3.3 (-3.9 to -2.7)	< 0.001
Duration of Mechanical Ventilation (days), mean ± SD	4.1 ± 1.9	6.8 ± 2.5	-2.7 (-3.3 to -2.1)	< 0.001
Time to First Defecation (days), mean ± SD	3.1 ± 0.8	5.2 ± 1.1	-2.1 (-2.4 to -1.8)	< 0.001
Mortality, *n* (%)				
In-hospital Mortality	4 (2.0%)	11 (5.5%)	-3.5% (RD)	0.074
Mortality after discharge (up to 1 year)	1 (0.5%)	4 (2.0%)	-1.5% (RD)	0.176
Invasive Interventions, *n* (%)				
Percutaneous Catheter Drainage	45 (22.5%)	48 (24.0%)	-1.5% (RD)	0.724
Endoscopic/Surgical Necrosectomy	12 (6.0%)	14 (7.0%)	-1.0% (RD)	0.686

Continuous variables were analyzed using linear regression

*Abbreviations*: *ERN* Early Rehabilitation Nursing, *SD* Standard Deviation, *CI* Confidence Interval, *aMD* adjusted Mean Difference, *ICU* Intensive Care Unit, *RD* Risk Difference

^*^The adjusted Mean Difference (aMD) for Hospital Length of Stay was calculated using a multivariate linear regression model adjusting for continuous baseline age and APACHE II scores

### Secondary outcomes

#### Mobilization level during ICU stay

The ERN group achieved significantly higher levels of mobilization compared to the control group throughout the first 14 days of the ICU stay. The mean highest ICU Mobility Scale (IMS) score was consistently higher in the ERN group, reaching a peak of 7.2 ± 1.5 compared to 4.1 ± 1.2 in the control group by day 14 (*P* < 0.001) (Supplementary Fig. 1).

#### Inflammatory response and muscle strength

At day 7 of intervention, the ERN group showed significantly lower serum levels of both CRP and IL-6 compared to the control group after adjusting for baseline levels (Table 3). At ICU discharge, patients in the ERN group had significantly higher MRC sum scores, with a lower incidence of ICU-AW (MRC score < 48) than the control group (18.5% vs. 45.0%, *P* < 0.001) (Table 3).

**Table 3:** Inflammatory markers, muscle strength, and safety outcomes

Parameter	ERN Group (*n* = 200)	Control Group (*n* = 200)	*P*-value
Inflammatory Markers at Day 7, mean ± SD			
CRP (mg/L)	48.5 ± 15.2	95.3 ± 25.4	< 0.001
IL-6 (pg/mL)	55.2 ± 18.3	110.6 ± 30.1	< 0.001
Outcomes at ICU Discharge			
MRC Sum Score, mean ± SD	52.4 ± 4.9	46.1 ± 5.8	< 0.001
Incidence of ICU-AW (MRC < 48), *n* (%)	37 (18.5%)	90 (45.0%)	< 0.001
Safety Outcomes & Complications, *n* (%)			
Ventilator-associated pneumonia	4 (2.0%)	22 (11.0%)	< 0.001
Pressure Ulcer (Stage ≥ 2)	2 (1.0%)	15 (7.5%)	0.001
Deep Vein Thrombosis	5 (2.5%)	15 (7.5%)	0.024
Total Complication Incidence	11 (5.5%)	52 (26.0%)	< 0.001
Adverse Events During Rehabilitation Sessions (ERN Group Only)			
Session Interruption due to Instability (Overall)	48 sessions (3.8%)	N/A	N/A
- In patients on MV	35 sessions (4.5%)	N/A	N/A
- In patients on CRRT	5 sessions (3.2%)	N/A	N/A
Device Dislodgement (CVC, ETT, NGT)	0 (0.0%)	N/A	N/A

Day 7 inflammatory markers were compared using ANCOVA, adjusting for baseline values

*Abbreviations*: *ERN* Early Rehabilitation Nursing, *SD* Standard Deviation, *CRP* C-Reactive Protein, *IL-6* Interleukin-6, *ICU* Intensive Care Unit, *MRC* Medical Research Council, *ICU-AW* ICU-Acquired Weakness, *MV* Mechanical Ventilation, *CRRT* Continuous Renal Replacement Therapy, *CVC* Central Venous Catheter, *ETT* Endotracheal Tube, *NGT* Nasogastric Tube, *N/A* Not Applicable

#### Long-term functional status and quality of life

The linear mixed-effects model revealed a significant group-by-time interaction for both ADL (*P* < 0.001) and all four domains of the WHOQOL-BREF (*P* < 0.001). While both groups showed improvement over time, the ERN group demonstrated a significantly greater and more sustained improvement in functional independence and quality of life at discharge, 3, 6, and 12 months (Table 4).

**Table 4:** Longitudinal assessment of ADL and QoL scores (linear mixed-effects model)

Outcome Measure	Group	Baseline	Discharge	3 Months	6 Months	12 Months	Coefficient (β)	SE	95% CI	*P*-value
ADL Score (0-100)	ERN (*n* = 188*)	35.4 ± 8.2	75.8 ± 8.1	85.2 ± 7.5	92.1 ± 6.9	96.5 ± 5.4	12.4	1.1	10.2 to 14.6	< 0.001
	Control (*n* = 180*)	36.1 ± 8.5	62.5 ± 9.3	70.3 ± 8.8	78.4 ± 9.1	83.2 ± 9.9				
QoL - Physical Health (0-100)	ERN (*n* = 188*)	38.2 ± 9.1	68.5 ± 10.2	78.3 ± 9.5	85.4 ± 8.2	90.1 ± 7.6	11.8	1.3	9.2 to 14.4	< 0.001
	Control (*n* = 180*)	38.9 ± 9.5	55.1 ± 11.4	63.2 ± 10.8	70.5 ± 10.1	75.3 ± 11.2				

Data are presented as mean ± SD

*Abbreviations*: *ADL* Activities of Daily Living, *QoL* Quality of Life, *ERN* Early Rehabilitation Nursing, *SE* Standard Error, *CI* Confidence Interval

*P*-value for group-by-time interaction was < 0.001 for both ADL and QoL-Physical Health. *Number of patients who completed the 12-month follow-up

#### One-year survival

**Fig. 2:**
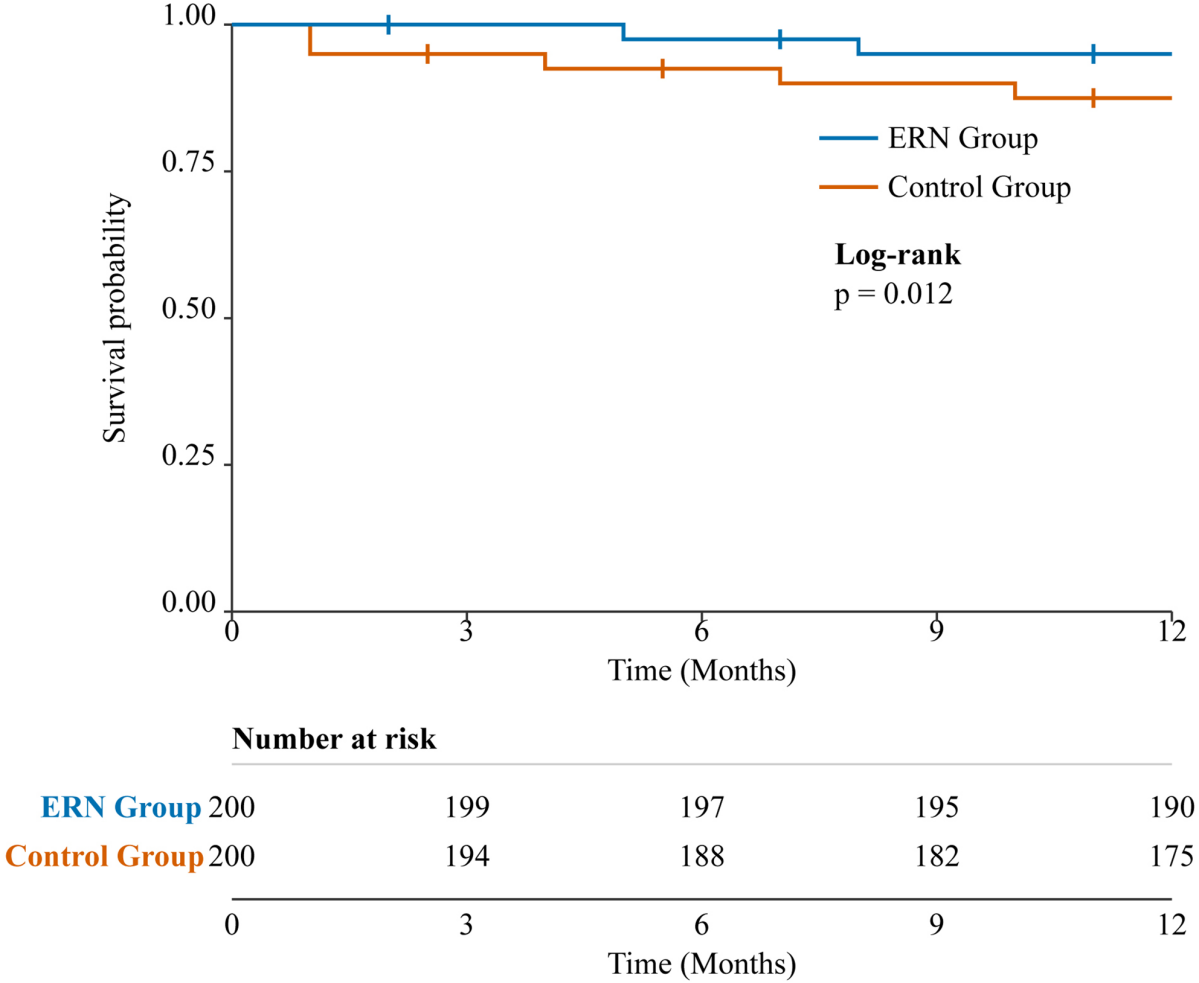
Kaplan-Meier Curves for 1-Year All-Cause Mortality. The ERN group had a significantly higher survival probability compared to the control group. The number of patients at risk is shown below the graph. *P*-value was calculated using the log-rank test. Abbreviations: ERN, Early Rehabilitation Nursing

A total of 15 patients (7.5%) in the control group and 5 patients (2.5%) in the ERN group died within the first year after randomization. The Kaplan-Meier analysis showed that the 1-year survival rate was significantly higher in the ERN group (95.0%) compared to the control group (87.5%) (log-rank test, *P* = 0.012). The adjusted hazard ratio (aHR) for 1-year mortality, controlling for age and APACHE II score, was 0.35 (95% CI, 0.14 to 0.88; *P* = 0.026) (Fig. 2).

#### Subgroup and safety analysis

**Fig. 3:**
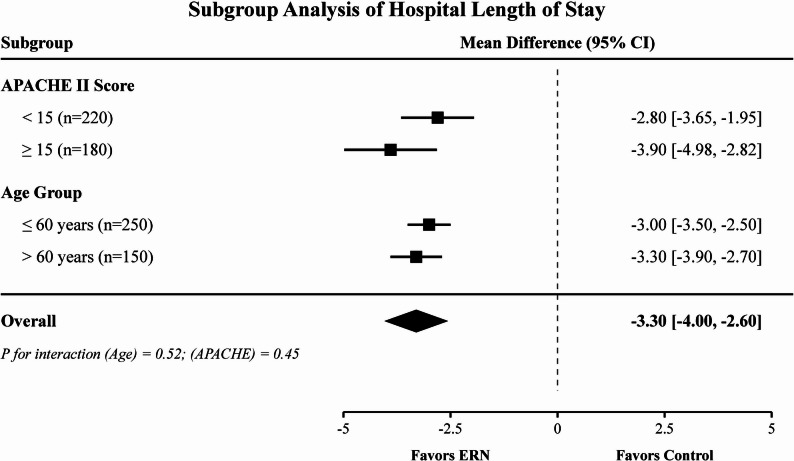
Forest plot of subgroup analysis for hospital length of stay. The effect of the early rehabilitation nursing (ERN) program was consistent across subgroups based on baseline APACHE II score and Age (≤ 60 years vs. >60 years). The overall estimate favors the ERN group. Squares represent the mean difference, and horizontal lines represent the 95% confidence intervals (CIs). Abbreviations: APACHE II, Acute Physiology and Chronic Health Evaluation II; ERN, Early Rehabilitation Nursing; CI, Confidence Interval

The pre-planned subgroup analysis showed that the beneficial effect of ERN on hospital LOS was consistent across patients with both lower (APACHE II < 15) and higher (APACHE II ≥ 15) disease severity, as well as across age groups (≤ 60 years vs. >60 years), with no significant interaction (P for interaction = 0.45 and 0.52, respectively) (Fig. 3). The overall incidence of evaluated ICU-acquired complications (ventilator-associated pneumonia, pressure ulcers, and deep vein thrombosis) was significantly lower in the ERN group (5.5% vs. 26.0%, *P* < 0.001) (Table 3). Adverse events during rehabilitation were rare. In patients receiving mechanical ventilation, 4.5% of sessions were interrupted due to physiological instability, compared to 3.2% in patients on CRRT. No accidental removal of central venous catheters or orotracheal tubes occurred during the sessions (Table 3). Patient satisfaction was also significantly higher in the ERN group, with 95.5% reporting being “satisfied” or “very satisfied” compared to 78.5% in the control group (*P* < 0.001).

## Discussion

This large randomized controlled trial provides robust evidence that a structured, multidisciplinary early rehabilitation nursing program significantly improves a wide spectrum of outcomes for patients with SAP. Our findings demonstrate that compared to usual care, the ERN program not only accelerates recovery and shortens ICU and hospital stays, but also attenuates the systemic inflammatory response, preserves muscle function, and, most importantly, leads to better long-term functional independence, quality of life, and a significant survival advantage at one year. These results address a critical gap in SAP management, shifting the paradigm from purely supportive care towards a proactive, restorative approach.

A key finding of our study is the profound impact of ERN on both short-term and long-term physical function. The 3.3-day reduction in both ICU and hospital stay is clinically meaningful and consistent with findings from general ICU populations [27], but our study is one of the largest to confirm this benefit specifically in SAP patients. While some large multicenter RCTs in general ICU populations have reported neutral findings on mortality and length of stay, our positive results may be explained by the comprehensive, protocolized, and multidisciplinary nature of our intervention, which contrasts with less standardized approaches in other trials [28]. Furthermore, patients with SAP may represent a distinct ICU subpopulation uniquely poised to benefit from ERN due to its potential to modulate the severe systemic inflammation and gastrointestinal dysfunction characteristic of the disease. The mechanisms underlying these benefits are likely multifactorial. The observed attenuation of CRP and IL-6 suggests that early physical activity may exert a systemic anti-inflammatory effect, potentially by reducing immobility-induced cytokine production and improving gut motility, which is critical in mitigating bacterial translocation in SAP [29]. This anti-inflammatory effect, coupled with improved respiratory mechanics from targeted training—a strategy known to improve diaphragmatic strength and reduce weaning time in general ICU populations [30, 31]—likely contributed to the shorter duration of mechanical ventilation and faster overall recovery. Furthermore, the preservation of muscle strength, evidenced by higher MRC scores and a 59% relative risk reduction in ICU-AW, is a crucial finding. Furthermore, it must be acknowledged that the ERN group was discharged from the ICU 3.3 days earlier on average; this shorter duration of profound immobilization likely acted synergistically with the active exercise to preserve muscle strength. ICU-AW is a debilitating consequence of critical illness that predicts long-term physical disability [32]. By actively combating immobility, our ERN program directly addressed the root cause of muscle wasting, which translated into superior ADL scores that were sustained up to one year post-discharge.

Perhaps the most significant contribution of our study is the demonstration of long-term benefits, including improved QoL and survival. Previous studies on severe acute pancreatitis (SAP) rehabilitation have rarely extended follow-up beyond hospital discharge, with significant heterogeneity in quality-of-life assessment methodologies and inconsistent reporting of long-term outcomes [17, 26]. More recent evidence confirms persistent knowledge gaps regarding standardized long-term functional recovery metrics beyond initial hospitalization [33]. Our 12-month data clearly show that the advantages gained in the hospital are not transient. The ERN group reported clinically and statistically significant higher scores across all QoL domains, particularly in physical health. The sustained benefits observed at 12 months, despite the lack of protocolized post-ICU rehabilitation, suggest that the ‘head start’ gained during the ICU stay—preventing severe muscle wasting and deconditioning—sets patients on a more positive recovery trajectory. Additionally, the educational component of the ERN program likely empowered patients to continue self-directed rehabilitation after discharge [34]. The observed 7.5% absolute improvement in 1-year survival is a striking finding. This survival benefit is likely mediated by the significant reduction in acute complications observed in our study (such as pneumonia and thrombosis), alongside the prevention of severe deconditioning, and potentially improved physiological reserve to handle future health stressors [35, 36]. Our safety analysis confirms that early rehabilitation is feasible even in critically ill SAP patients, with a very low incidence of adverse events or device dislodgements (Table 3), supporting the safety profile of our protocol.

Our study is strengthened by its randomized design, large sample size, low attrition rate, long-term follow-up, and the use of a wide range of validated, patient-centered outcomes. The protocolized nature of our ERN program ensures its reproducibility in other centers. However, we acknowledge several limitations. First, this was a single-center study conducted at a tertiary academic medical center with a dedicated rehabilitation team, which may limit the generalizability of our findings to other settings with different resources or expertise. Therefore, multi-center trials are needed to confirm these results before ERN can be universally recommended as a standard of care. Second, due to the nature of the intervention, blinding of clinicians and patients was not possible, which could introduce performance bias. We mitigated this by using blinded outcome assessors for all key endpoints. Third, while we tracked inflammatory markers, we did not measure more specific markers of muscle catabolism or anabolic hormones, which could have provided deeper mechanistic insights. Fourth, while we used blinded assessors for functional outcomes, the non-blinding of patients and clinical staff could have introduced a performance bias or a Hawthorne effect, potentially influencing subjective outcomes like patient satisfaction and QoL. Fifth, we did not systematically inquire about or track the specific physical activities or outpatient rehabilitation services patients pursued after hospital discharge. The lack of tracking post-discharge interventions means we cannot exclude the possibility that differential access to or engagement in community rehabilitation influenced the long-term functional and quality of life outcomes. Finally, while we assumed pre-morbid muscle strength was similar due to randomization, the lack of pre-ICU baseline strength data is a limitation.

In conclusion, our study provides compelling evidence that a structured early rehabilitation nursing program is a safe and highly effective intervention for patients with SAP. It reduces the burden of critical illness by decreasing length of stay and complications, while profoundly improving long-term physical function, quality of life, and survival. These findings strongly advocate for the integration of early, protocol-based rehabilitation as a standard of care in ICUs managing this challenging patient population.

## Supplementary Information

Supplementary Material 1. Supplementary Figure 1: Daily Highest Achieved ICU Mobility Scale (IMS) Score. Line graph showing the mean daily highest IMS score for the ERN and control groups during the first 14 days of ICU stay. The ERN group demonstrated consistently higher mobilization levels. Shaded areas represent 95% confidence intervals. Data were analyzed using a linear mixed-effects model. The reported P-value (<0.001) represents the group-by-time interaction effect. The table below the x-axis indicates the number of patients remaining in the ICU at each time point. Abbreviations: ICU, Intensive Care Unit; ERN, Early Rehabilitation Nursing.

Supplementary Material 2. Supplementary Table 1: Detailed Protocol of the Structured Early Rehabilitation Nursing (ERN) Program.

## Data Availability

The data and materials used and/or analysed during the current study are available from the corresponding author on reasonable request.

## Abbreviations

ADL: Activities of Daily Living
CRP: C-Reactive Protein
ERN: Early Rehabilitation Nursing
ICU: Intensive Care Unit
ICU-AW: ICU-Acquired Weakness
IL-6: Interleukin-6
LOS: Length of Stay
MRC: Medical Research Council
QoL: Quality of Life
SAP: Severe Acute Pancreatitis
