# Supplementary figures and images for: Effect of a structured early rehabilitation program on long-term functional recovery, quality of life, and survival in patients with severe acute pancreatitis: a randomized controlled trial
Source: BMC Gastroenterol. 2026 May 27;26:471. doi: 10.1186/s12876-026-04955-7 (PMC13397734; doi:10.1186/s12876-026-04955-7)

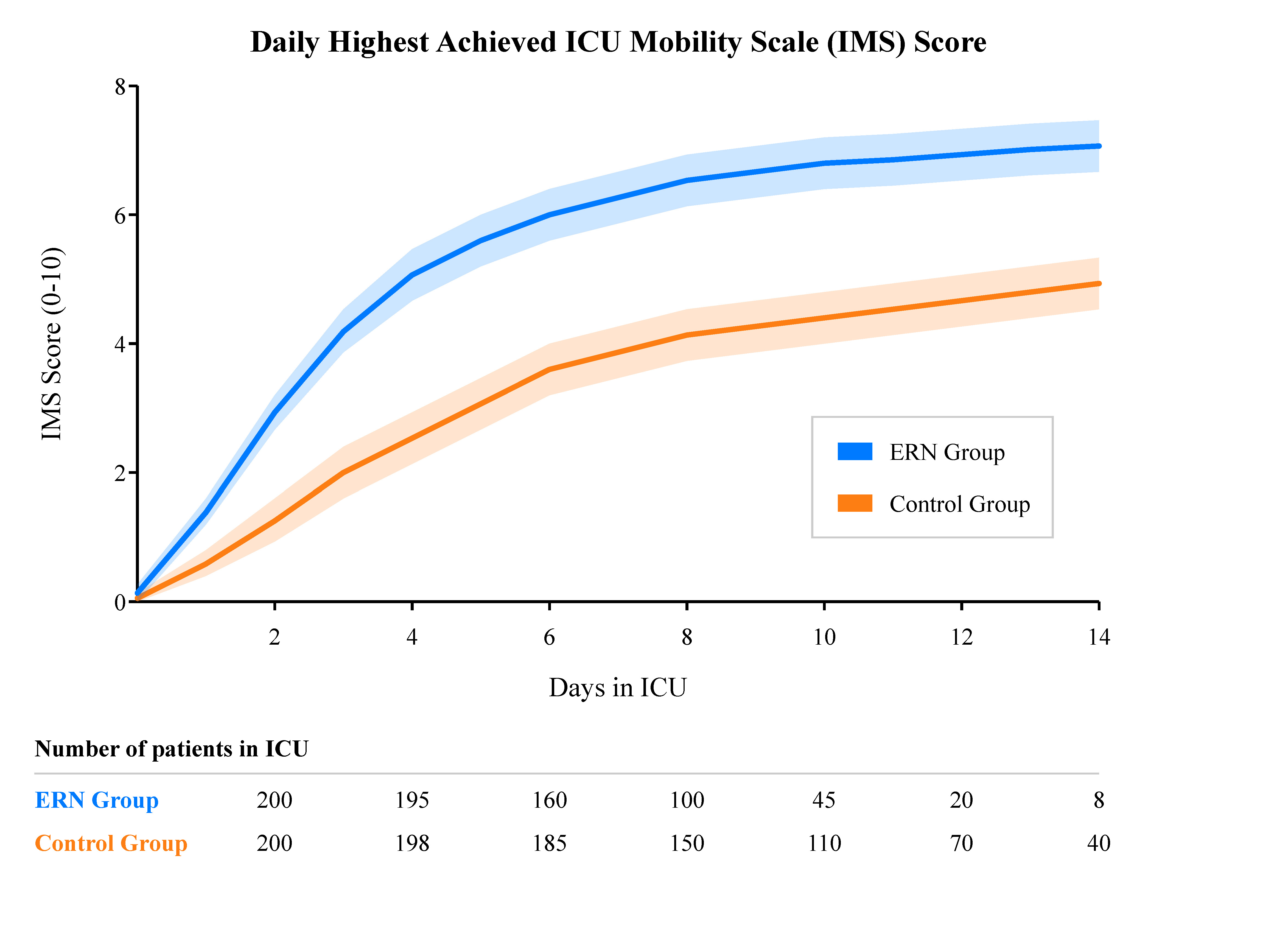

Supplement: Supplementary file 1 — Supplementary Material 1. Supplementary Figure 1: Daily Highest Achieved ICU Mobility Scale (IMS) Score. Line graph showing the mean daily highest IMS score for the ERN and control groups during the first 14 days of ICU stay. The ERN group demonstrated consistently higher mobilization levels. Shaded areas represent 95% confidence intervals. Data were analyzed using a linear mixed-effects model. The reported P-value (<0.001) represents the group-by-time interaction effect. The table below the x-axis indicates the number of patients remaining in the ICU at each time point. Abbreviations: ICU, Intensive Care Unit; ERN, Early Rehabilitation Nursing. [file 12876_2026_4955_MOESM1_ESM.jpg]
