# Supplementary material for: Effect of a structured early rehabilitation program on long-term functional recovery, quality of life, and survival in patients with severe acute pancreatitis: a randomized controlled trial
Source: BMC Gastroenterol. 2026 May 27;26:471. doi: 10.1186/s12876-026-04955-7 (PMC13397734; doi:10.1186/s12876-026-04955-7)
Supplement: Supplementary file 2 — Supplementary Material 2. Supplementary Table 1: Detailed Protocol of the Structured Early Rehabilitation Nursing (ERN) Program. [file 12876_2026_4955_MOESM2_ESM.docx]

**Supplementary Table 1. Detailed Protocol of the Structured Early Rehabilitation Nursing (ERN) Program.**

| **Component** | **Phase 1: Unconscious/Deeply Sedated** | **Phase 2: Awake & Hemodynamically Stable** | **Phase 3: Active Mobilization** |
| --- | --- | --- | --- |
| **Progression Criteria** | N/A (Entry phase) | RASS -2 to +1; MAP ≥65 mmHg without high-dose vasopressors (defined as norepinephrine equivalent dose < 0.1 μg/kg/min); FiO2 ≤0.6, PEEP ≤10 cmH2O. | Tolerates sitting at edge of bed for ≥20 mins; MRC score ≥3 in anti-gravity muscles; able to follow commands. |
| **Mobilization** | Passive range of motion (PROM) all limbs (20 mins, 2x/day). Regular repositioning (q2h). | Active-assisted/active ROM. Bridging, rolling. Head of bed elevation. Sitting at edge of bed (1-2x/day, 20-30 mins). Transfer to chair. | In-place stepping. Supervised ambulation with walker (goal: ≥10 meters, 2-3x/day). |
| **Respiratory Therapy** | Chest physiotherapy as indicated. | Deep breathing exercises. Incentive spirometry (10 breaths/hour while awake). Cough assistance. | Coordinated breathing with ambulation. Continued spirometry. |
| **Psychological Support** | Structured orienting communication by staff (5 mins, 2x/day). | Standardized protocol (15 mins/day) by trained nurses: 5 mins orienting communication, 5 mins systematic goal-setting, 5 mins progressive relaxation/deep-breathing for anxiety management. Family engagement. | Continued structured support (15 mins/day), reinforcement of progress, standardized preparation for discharge. |
| **Safety Monitoring & Stopping Criteria** | Continuous monitoring. Stop/Pause session for: HR <40 or >130 bpm or >20% change; SBP <90 or >180 mmHg; SpO2 <90%; new arrhythmia; signs of respiratory distress; patient distress/pain. | | |

Note: RASS, Richmond Agitation-Sedation Scale; MAP, Mean Arterial Pressure; FiO2, Fraction of Inspired Oxygen; PEEP, Positive End-Expiratory Pressure; MRC, Medical Research Council; ERN, Early Rehabilitation Nursing; PROM, Passive Range of Motion; ROM, Range of Motion; HR, Heart Rate; SBP, Systolic Blood Pressure; SpO2, Peripheral Capillary Oxygen Saturation.
